# P130cas is required for TGF-β1-mediated epithelial-mesenchymal transition in lung cancer

**DOI:** 10.3892/ol.2014.2123

**Published:** 2014-05-08

**Authors:** BO DENG, QUN-YOU TAN, RU-WEN WANG, YAO-GUANG JIANG, JING-HAI ZHOU, WEI HUANG

**Affiliations:** Department of Thoracic Surgery, Institute of Surgery Research, Daping Hospital, Third Military Medical University, Chongqing 400042, P.R. China

**Keywords:** P130cas, breast cancer anti-estrogen resistance protein 1, p38, TGF-β1, non-small cell lung cancer, epithelial-mesenchymal transition

## Abstract

In lung cancer A549 cells, the present study evaluated the associations between p130cas expression and the activation of p38 or Smad2, which are components of two of the main signaling pathways of transforming growth factor-β1 (TGF-β1), i.e., epithelial-mesenchymal transition (EMT) and apoptosis, respectively. TGF-β1-induced EMT was investigated by inspecting cell shape and cell migration, and by testing E-Cadherin, N-Cadherin and Vimentin biomarkers in p130cas-RNA interference (RNAi)-A549 cells. The changes in TGF-β1-induced apoptosis, i.e., cleaved Caspase-3 levels, were additionally analyzed following p130cas-RNAi. p130cas-knockdown decreased the phosphorylated (p)-p38 expression level, and blockaded the TGF-β1-induced activation of p-p38 in the A549 cells. p130cas-knockdown arrested cell migration and impaired TGF-β1-induced EMT in the A549 cells, characterized by changes in cell morphology and biomarker levels. However, p130cas-knockdown had no impact on the activation of Smad2 and the cleavage of Caspase-3. These results indicate that p130cas is a novel molecular ‘rheostat’ that alters the function of the TGF-β1 signaling pathway from tumor suppression to tumor promotion in lung cancer cells. The underlying mechanism warrants further study.

## Introduction

p130cas, also known as breast cancer anti-estrogen resistance 1, is one of the Crk-associated substrate (cas) protein family members. The designation Crk stands for CT10 regulator of kinase, where CT10 is the avian virus from which a protein was isolated, without kinase domains, but able to stimulate tyrosine phosphorylation in cells (1). p130cas was originally identified as a cellular protein migrating at 130 kDa of molecular weight, and was hyperphosphorylated in v-Crk- and v-Src-transformed cells (2). Our recent pilot studies indicated the clinical and biological implications of p130cas in non-small cell lung cancer (NSCLC) (3, 4). Serum p130cas levels were significantly higher in NSCLC compared with the control group, gradually increasing with the progression of tumor staging, and decreasing following malignant lesion removal (3). In a cohort of 151 Chinese patients with NSCLC, elevated p130cas protein expression levels in tumor tissues were shown to predict a poor prognosis (hazard ratio, 1.777; P=0.028) (4). In addition, p130cas-knockdown caused cell migration inhibition and arrest of cell growth and the cell cycle in A549 lung cancer cells (4).

Elevating the expression of either the full-length or just the carboxyl terminus of p130Cas in mammary epithelial cells has been shown to diminish the ability of transforming growth factor-β1 (TGF-β1) to activate Smad2/3, but increase its coupling to p38 mitogen-activated protein kinases (MAPKs; p38) (4,5), whose activation is required for TGF-β1 mediated fibroblastic transdifferentiation and cell migration (6). Moreover, p38 inhibitors can block TGF-β1-induced epithelial-mesenchymal transition (EMT) in a variety of cells (6,7), including pulmonary epithelial cells (7). The p38 signaling cascade has significant roles in TGF-β1-induced metastasis, particularly in the late stages of malignancy (8,9).

Our previous study demonstrated that p130cas-knockdown reduced the phosphorylated-p38 (p-p38) level in lung cancer A549 cells (4), indicating the significance of p130cas in p38 activation and the subsequent occurrence of EMT.

The present study evaluated the correlations between p130cas-expression and the activation of p38 and Smad2, which are components of the two main signaling pathways of TGF-β1, i.e., EMT and apoptosis (10,11). The aim of the current study was to clarify the critical roles of p130cas in the regulation of TGF-β1-mediated EMT or apoptosis in A549 cells. Additionally, the aim was to clarify the significant alteration of the TGF-β1 signaling pathways from tumor suppression to tumor promotion by the regulation of p130cas.

## Materials and methods

### Cell culture and RNA interference (RNAi) of p130cas in A549 cells

The A549 lung adenocarcinoma cell line was obtained from the American Type Culture Collection (Manassas, VA, USA) and cultured in RPMI 1640/10% fetal bovine serum.

The RNAi p130cas protocol was established according to our previous published study (4). The following oligoribonucleotide pairs were used: 5′-CCGGGG TCGACAGTGGTGTGTATTTCAAGAGAATACACACCAC TGTCGACCTTTTTTg-3′ and 5′-AATTCAAAAAAGGTC GACAGTGGTGTGTATTCTCTTGAAATACACACCACT GTCGACC-3′. Entire sequences were derived from the sequence of human p130cas mRNA. The oligonucleotides were obtained from Sunbio Medical Biotechnology Co., Ltd. (Shanghai, China). The two complementary strands (each 20 mM) in 60 ml annealing buffer (Sunbio Medical Biotechnology Co., Ltd.) were heated for 5 min at 95°C and then incubated for 1 h at room temperature. Thereafter, the green fluorescent protein (GFP)-tagged lentiviral vector, pLVT351.LV for p130cas-RNAi, was constructed by inserting the annealing nucleotides into the *Age*I+*Eco*RI site of pMAGic 4.1 (Sunbio Medical Biotechnology Co., Ltd.).

Secondly, the A549 cell line was plated at 2.3×10^5^ cells per well in a 24-well culture plate and infected with lentivirus at a multiplicity of infection value of 10. The cells infected with pLVT351.LV and CMV-GFP-LV (blank lentiviral vector; pMAGic 4.1) were termed the A549-p130cas-RNAi and A549 negative control cells, respectively. The efficacy of the RNAi of p130cas was confirmed by our previous study (4).

### Quantitative reverse transcription polymerase chain reaction (RT-qPCR)

In order to evaluate the efficacy of p130cas RNAi, RT-qPCR was performed in A549-p130cas-RNAi and A549-negative control cells. The cells were seeded at a concentration of 1×10^5^ cells/well in 6-well plates. Two days after seeding, total RNA was extracted from the cells using TRIzol (Invitrogen Life Technologies, Carlsbad, CA, USA). First-strand cDNA was synthesized with M-MLV transcriptase (Promega, Madison, WI, USA) and oligo dT. RT-qPCR was performed using SYBR Green PCR master mix (Takara Bio, Inc., Shiga, Japan) and the ABI Prism 7000 sequence detection system (Applied Biosystems, Foster City, CA, USA). The following PCR primers were used: 5′-CAATGCCTCACTGCTCTT-3′ and 5′-GTAGTCATAGTCCTCCATC-3′. The specificity of detected signals was confirmed by a dissociation curve consisting of a single peak. All samples were performed in duplicate. Values were normalized by human β-actin.

### Cytokine treatment and cell morphology inspection

The A549 cells were co-incubated at 37°C in a humidified atmosphere of 5% CO_2_ with 7.5 ng/ml TGF-β1 (catalog no. 01-209; Upstate Biotechnology, New York, NY, USA) for 1, 12 and 36 h. Cell morphology was analyzed using light microscopy, focusing on the changes of colonial morphology and lamellipodia.

### Cell invasiveness and migration assay

For the assessment of cell motility, chamber invasiveness and migration assays were conducted using a cell culture insert (8-mm pore size, 24-well format; Cell Invasion Assay kit, cat. no. ECM550; Chemicon, Temecula, CA, USA). The cells were seeded in duplicate at a density of 3.0×10^5^ cells/chamber. After 48 h, the cells which had not moved to the lower wells were removed from the upper face of the filters using cotton swabs, and the cells that had moved to the lower surface of the filter were stained using a cell invasion assay kit, which utilizes ECMatrix™, a reconstituted basement membrane matrix of proteins derived from the Engelbreth Holm-Swarm mouse tumor. The cells were quantified by visual counting once images had been captured. Experiments were performed in triplicate. Mean values for three random fields were obtained for each well.

### Immunoblotting of biomarkers

A549 cell lysate was prepared by homogenization in a RIPA buffer comprised of 50 mM Tris-HCl (pH 7.5), 150 mM NaCl, 1% Triton X-100, 0.1% sodium dodecyl sulfate (SDS), 0.5% deoxycholic acid and 0.02% sodium azide. The protein concentrations were determined with a Bicinchoninic Acid Protein Assay kit (Pierce, Rockford, IL, USA). Proteins were denatured at 95°C for 5 min, and 50 mg protein per lane was resolved by SDS-polyacrylamide gel electrophoresis using 10% polyacrylamide gel. Proteins were blotted on polyvinylidene difluoride membranes (Thermo Fisher Scientific, Waltham, MA, USA), which were then blocked with 5% skimmed milk for 1 h at room temperature. The proteins were immunoblotted using anti-E-cadherin antibody (1:1,000; Cell Signaling Technology, Inc., Danvers, MA, USA), anti-N-cadherin antibody (1:1,000; Cell Signaling Technology, Inc.), anti-Vimentin antibody (1:200; Sigma Aldrich, St. Louis, MO, USA), anti-Caspase-3 (Cleaved-Asp175) antibody (1:1,000; Assay Biotechnology, Co., Inc., Sunnyvale, CA, USA), anti-p-p38 antibody (Thr180/Tyr182; 1:1,000; Cell Signaling Technology, Inc.), anti-p38 antibody (1:1,000; BD Transduction Laboratories, BD Biosciences, Franklin Lakes, NJ, USA), anti-smad2 antibody (1:500; Abcam, Cambridge, MA, USA), anti-p-Smad2 antibody (Ser467; 1:1,000; Bioworld Technology, St. Louis Park, MN, USA), anti-p130cas antibody (1:1,000; BD Transduction Laboratories, BD Biosciences) and anti-p-p130cas (Tyr165; 1:1,000; Cell Signaling Technology, Inc.). An anti-GAPDH (Sigma Aldrich) antibody served as the control. Experiments were performed in triplicate. Immunoblotting was quantified with Quantity One software (Bio-Rad, Hercules, CA, USA). The analyses of the bands of the different proteins were referenced against GAPDH.

### Data analysis

The statistical analysis of the association between the protein levels was performed using Pearson’s correlation analysis. Student’s t-test was used to evaluate differences in the variables (mean ± standard error of the mean) between the two study groups. The analysis was performed using SPSS Version 11.0 software for Windows (SPSS, Inc., Chicago, IL, USA). P<0.05 (two-sided) was considered to indicate a statistically significant difference.

## Results

### p130cas-knockdown reduces p-p38 levels in A549 cells and blockades phosphorylation of p38 induced by TGF-β1, but has no impact on the activation of Smad2

**Figure 1:**
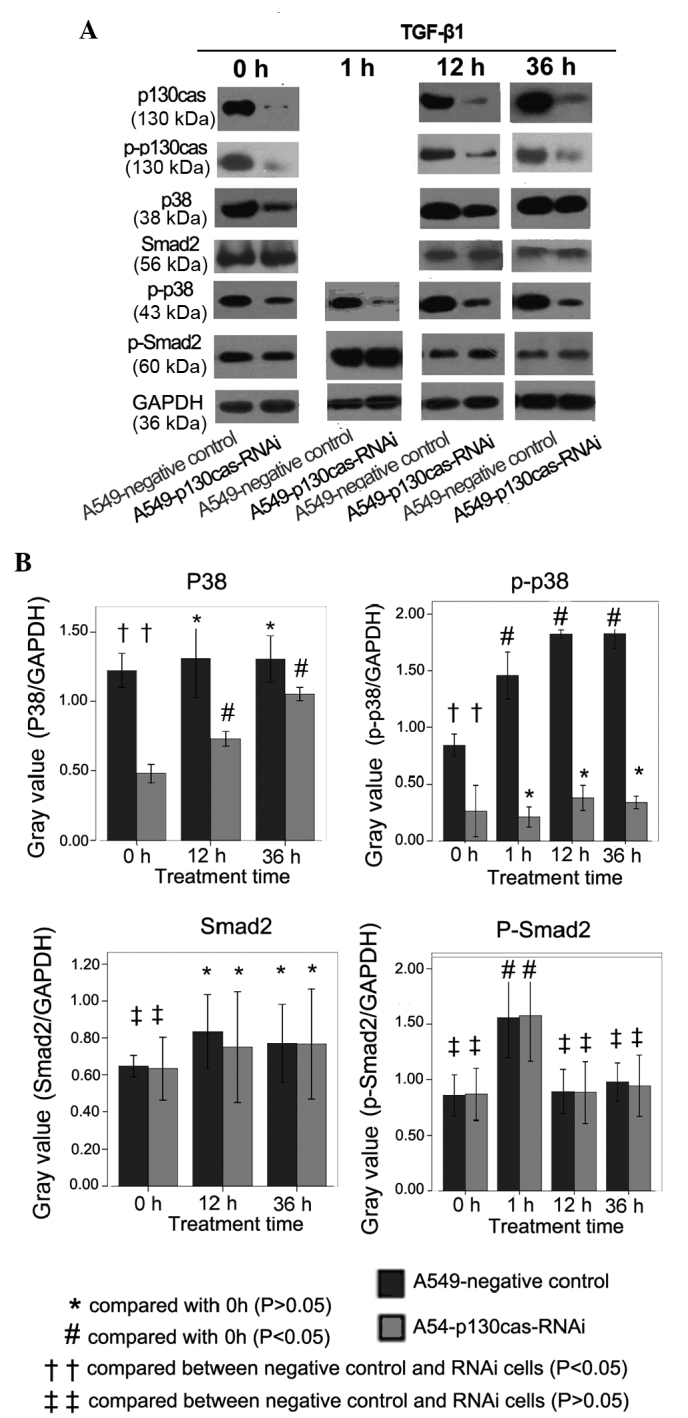
(A) Immunoblotting was conducted to detect the expression levels of p130cas, p-p130cas, p38, p-p38, Smad2 and p-Smad2. The expression levels of p130cas-protein and p-p130cas were reduced markedly in the A549-p130cas-RNAi cells compared with the negative control cells. TGF-β1 treatment (7.5 ng/ml) for 12 h or 36 h markedly increased the p130cas expression levels in the A549 cells. The expression levels of total-p38 and p-p38 were reduced significantly in the A549-p130cas-RNAi cells compared with the A549-negative control cells. The total-p38 expression level was significantly upregulated in the A549-p130cas-RNAi cells following TGF-β1 treatment, however, the p-p38 expression level in the A549-p130cas-RNAi cells was consistently significantly lower than in the A549-negative control cells prior to or following TGF-β1 treatment. TGF-β1 remarkably increased the expression levels of p-Smad2 in the A549 cells following 1 h of treatment. However, p130cas-knockdown had no impact on the expression of total-Smad2 or p-Smad2 compared with the control cells. (B) Quantification of immunoblotting was conducted with Quantity One software. The analyses of the bands of the different proteins were reference against GAPDH. Mean ± SEM values were calculated in order to establish any statistically significant differences. TGF-β1, transforming growth factor-β1; RNAi, RNA interference; cas, Crk-associated substrate.

A549 cells that stably expressed pLVT351-LV (A549-p130cas-RNAi cells) and CMVGFP-LV (A549-negative control cells) were established through use of a lentivirus system (4). At least an 80% reduction in p130cas-mRNA and -protein in A549-p130cas-RNAi cells was confirmed by RT-qPCR (4), and western blotting analysis (Fig. 1A), respectively. Additionally, the expression level of p-p130cas was reduced markedly following p130cas-RNAi (Fig. 1A). TGF-β1 treatment markedly increased the expression level of p130cas in the A549-negative control cells and the A549-p130cas RNAi cells (Fig. 1A).

The expression levels of total-p38 and p-p38 were reduced significantly in the A549-p130cas-RNAi cells compared with the A549-negative control cells (P=0.02 and 0.01, respectively; Fig. 1A and B). Notably, the total-p38 expression level was significantly upregulated in the A549-p130cas-RNAi cells following TGF-β1 treatment (P=0.02; Fig. 1A and B), however, the p-p38 expression level in the A549-p130cas-RNAi cells was consistently significantly (P=0.01; Fig 1A and B) lower than in the A549-negative control cells prior to and following TGF-β1 treatment (P=0.008; Fig. 1A and B), indicating that the TGF-β1-induced phosphorylation of p38 was effectively blocked by p130cas-knockdown.

In addition, TGF-β1 markedly increased the expression levels of p-Smad2 in the A549-negative control cells and the A549-p130cas-RNAi cells following 1 h of treatment (Fig. 1A and B). However, p130cas-knockdown had no impact on the expression of total-Smad2 or p-Smad2 compared with the control cells (Fig. 1A and B).

### p130cas-knockdown arrests cell invasion and the EMT induced by TGF-β1 in A549 cells, but has no impact on the cleavage of Capase-3

Co-incubation of TGF-β1 and epithelial cells is a convenient way to induce EMT (11,12), which may be characterized by changes in cell shape, cell migration and biomarkers, e.g., the downregulation of E-Cadherin and the upregulation of N-Cadherin (13). Therefore, the following investigations were conducted in the present study in order to clarify the impact of p130cas on TGF-β1-induced EMT in A549 cells.

**Figure 2:**
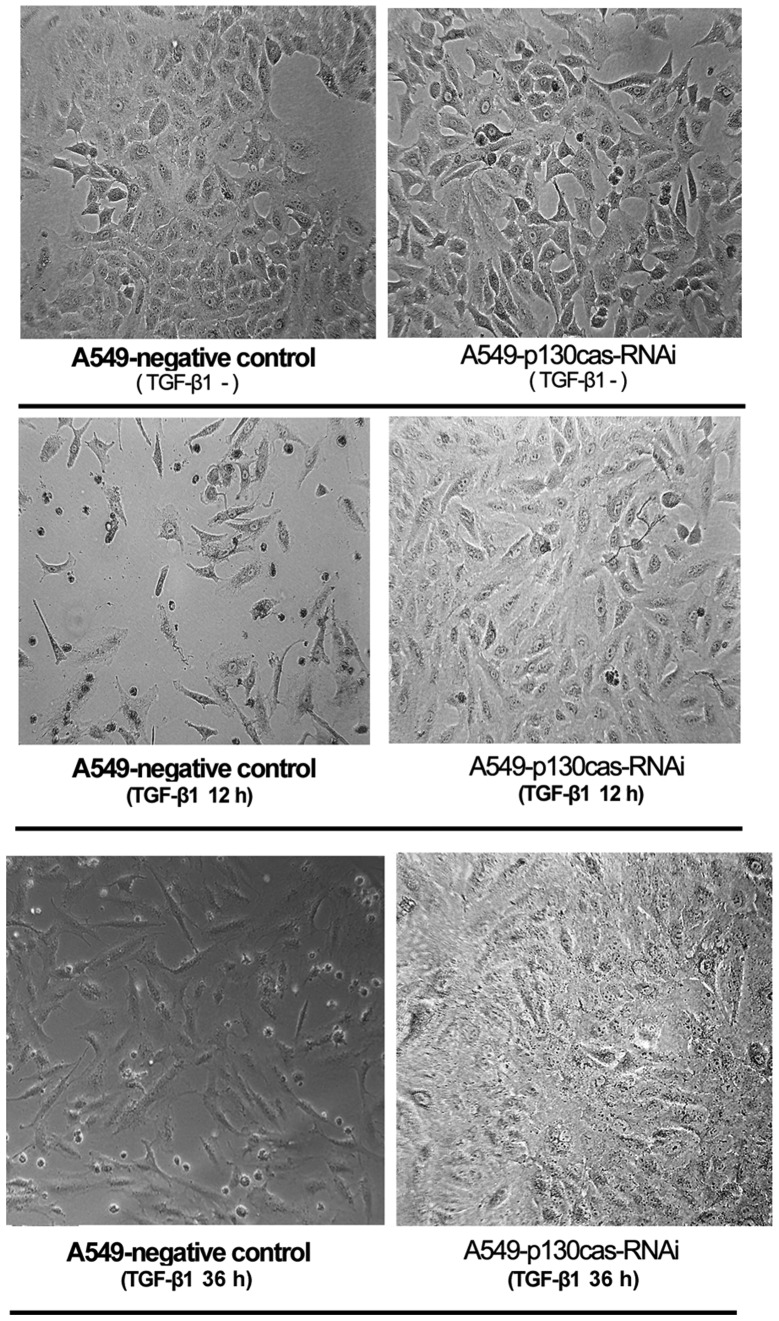
Cell morphology was observed using light microscopy. In the A549-negative control cells, TGF-β1 treatment (7.5ng/ml) for 12 h or 36 h induced EMT-related cell shapes, including the loss of colonial morphology and increased lamellipodia. TGF-β1 treatment did not induce these morphological changes in the A549-p130cas-RNAi cells (magnification, ×200). TGF-β1, transforming growth factor-β1; RNAi, RNA interference; cas, Crk-associated substrate.

In the A549-negative control cells, TGF-β1 treatment (12 h or 36 h) induced EMT-associated cell shapes, including loss of colonial morphology and increased lamellipodia (Fig. 2). However, TGF-β1 treatment did not induce these morphological changes in the A549-p130cas-RNAi cells (Fig. 2).

**Figure 3:**
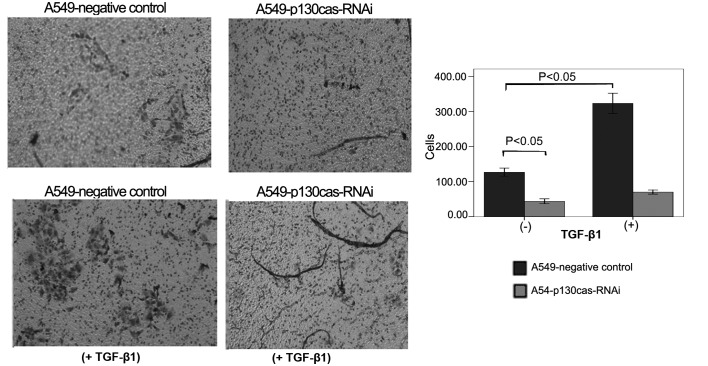
For the assessment of cell motility, chamber invasiveness and migration assays were conducted using a cell culture insert. The cells were seeded in duplicate at a density of 3.0×10^5^ cells/chamber. Subsequent to 48 h, the cells which had not moved to the lower wells were removed from the upper face of the filters using cotton swabs, and the cells that had moved to the lower surface of the filter were stained. The cells were quantified by visual counting. Mean values for three random fields were obtained for each well. Subsequent to 48 h of treatment, TGF-β1 markedly stimulated the migration of the A549-negative control cells (P<0.05) and the A549-p130cas-RNAi cells (P<0.05), respectively. However, the migrated cells of the p130cas-RNAi group were consistently significantly fewer in number compared with the negative control group prior to or following TGF-β1 treatment (P<0.05). TGF-β1, transforming growth factor-β1; RNAi, RNA interference; cas, Crk-associated substrate.

Subsequent to 48 h of treatment, TGF-β1 markedly stimulated the migration of the A549-negative control cells (130±10 vs. 320±25, P=0.0003; Fig. 3) and the A549-p130cas-RNAi cells (43±6 vs. 70±6, P=0.005; Fig. 3), respectively. However, the migrated cells of the p130cas-RNAi group were consistently significantly less in number compared with those of the negative control group prior to or following TGF-β1 treatment (43±6 vs. 130±10, P=0.0002; 70±6 vs. 320±25, P=0.0001; Fig. 3).

**Figure 4:**
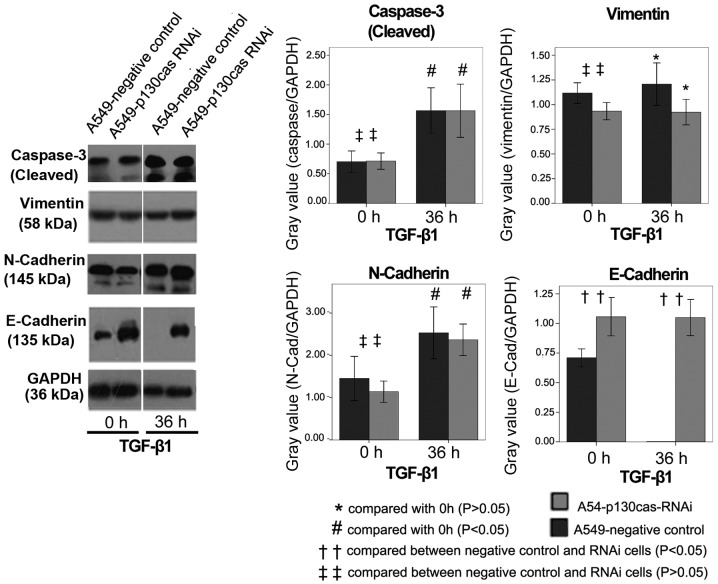
Immunoblotting was conducted to detect the levels of E-Cadherin, N-Cadherin, Vimentin and cleaved Caspase-3. Quantification of immunoblotting was conducted with Quantity One software. The analyses of the bands of the different proteins were referenced against GAPDH. Mean ± SEM values were calculated in order to establish any statistically significant differences. E-Cadherin expression levels were significantly higher after p130cas RNAi (P<0.05). Subsequent to 36 h of incubation with TGF-β1, E-Cadherin was consistently abundant in the A549-p130cas-RNAi cells, but undetectable in the A549-negative control cells. The N-cadherin levels were slightly lower in the A549-p130cas-RNAi cells than in the A549-negative control cells. TGF-β1 treatment markedly increased the N-Cadherin levels in the A549-p130cas-RNAi and A549-negative control cells following 36 h of treatment. There was no appreciable disparity in Vimentin levels prior to or following TGF-β1 treatment. The cleaved Caspase-3 levels were markedly increased in the A549-p130cas-RNAi cells and the A549-negative control cells following 36 h of TGF-β1 treatment. Significantly, p130cas-knockdown had no impact on the cleavage of Caspase-3 compared with the control cells. TGF-β1, transforming growth factor-β1; RNAi, RNA interference; cas, Crk-associated substrate.

E-Cadherin expression levels were significantly higher in A549-p130cas-RNAi cells than in A549-negative control cells (P=0.0001; Fig. 4). After 36 h incubation with TGF-β1, E-Cadherin was consistently abundant in A549-p130cas-RNAi cells, but undetectable in A549-negative control cells (Fig. 4). N-cadherin levels were slightly lower in A549-p130cas-RNAi cells than in A549-negative control cells (Figure 4). TGF-β1 remarkably increased N-Cadherin in either A549-p130cas-RNAi or negative control cells after 36 h treatment (Fig. 4). There was no evident disparity in Vimentin levels prior to or following TGF-β1 treatment (Fig. 4).

Cleaved Caspase-3 levels were markedly increased in the A549-p130cas-RNAi cells and negative control cells following 36 h of TGF-β1 treatment (Fig. 4). Significantly, p130cas-knockdown had no impact on the cleavage of Caspase-3 (P=0.82; Fig. 4) compared with the control cells.

## Discussion

A number of studies (14–16) have demonstrated evidence of EMT, which is a critical event in cancer progression, characterized by morphological changes, enhanced cell motility, downregulation of epithelial markers and up-regulation of mesenchymal markers (13), in a variety of malignancies. TGF-β1 has been found to be upregulated in a variety of tumors (17–19), and plays significant roles in EMT and metastasis (12). Co-incubation of TGF-β1 and epithelial cells is a convenient way to induce EMT in various cells (12). Thus far, the p38 pathway, also known as the non-canonical TGF-β1 signaling pathway (20), has been found to be critical in TGF-β1-mediated EMT (7), and p38 inhibitors have been shown to block TGF-β1-induced EMT in a variety of cell lines (6). Using immunoblotting, Greenberg *et al* (21) found that compared with normal tissue, only activated p38 MAPK was consistently increased in NSCLC, indicating that this pathway has additional roles in malignant cell growth or transformation. In our previous study, p130cas was shown to be critical in the activation of p38 *in vivo* and *in vitro* (4). To the best of our knowledge, few studies have focused on the issues analyzed in the present study of whether there is any association between p130cas expression and TGF-β1-induced EMT in lung cancer, and whether p130cas-knockdown is able to inhibit TGF-β1-induced EMT in lung cancer.

A549 lung cancer is a well-characterized cell line that has been used as a model system to study the underlying mechanisms of carcinogenesis, apoptosis and cancer progression in lung cancer (22). Moreover, A549 cells are conventionally used to study EMT (22). Hence, it was chosen as a model system for the present study.

In the A549-negative control cells, TGF-β1 treatment led to the marked elimination of E-Cadherin, which is the prototype epithelial cell marker of EMT, whose expression has been found to be downregulated markedly during EMT (23). Additionally, TGF-β1 treatment led to the increased expression of N-Cadherin, which is a mesenchymal marker (24), indicating the occurrence of EMT in the A549-negative control cells. However, TGF-β1 treatment did not result in any elimination of E-Cadherin expression in the A549-p130cas-RNAi cells. Meanwhile, the expression levels of N-Cadherin were slightly lower in the p130cas-RNAi cells compared with the control cells. All the results indicated that p130cas-knockdown resulted in the inhibition of EMT, leading to changes in the expression of epithelial and mesenchymal markers. Recently, Miao *et al* (25) also found that the overexpression of p130cas was significantly correlated with the decreased expression of E-cadherin in 105 NSCLC tissues (P=0.002). Although the study by Miao *et al* (*in vivo*) and the present study (*in vitro*) simultaneously supported that the significantly adverse association between the expression of p130cas and E-cadherin in NSCLC, the underlying mechanism remains unknown. Tikhmyanova and Golemis (26) found that cas proteins promoted the lysosomal degradation of E-cadherin via Src kinase in MCF7 breast adenocarcinoma cells. We hypothesize that there is similar mechanism for the p130cas-induced lysosomal degradation of E-cadherin in NSCLC, which warrants further study.

In the present study, the cell shapes of the A549-negative control cells, characterized by the loss of colonial morphology and increased lamellipodia, following TGF-β1 treatment strongly demonstrated the occurrence of EMT. However, TGF-β1 did not stimulate EMT-associated morphological changes in the p130cas-RNAi cells. Moreover, Transwell cell assays supported the fact that p130cas-knockdown significantly blocked the enhancement of cellular motility induced by TGF-β1.

Therefore, p130cas has been shown to be required for TGF-β1-mediated EMT in A549 cells. However, the underlying mechanisms warrant further investigations. The present study indicated that p130cas-knockdown decreased the expression level of p-p38 in the A549 cells and blockaded the TGF-β1-induced activation of p-p38. The p38 MAPK signaling pathway has been found to be involved in the phosphorylation of Twist1, the stabilization of Twist1 protein and the induction of EMT in mammary epithelial cells (27). In addition, the close correlations between Snail (an E-Cadherin repressor) and phospho-p38 expression levels have been confirmed in ovarian carcinoma, demonstrating the ability of p38 MAPK to induce EMT via Snail (28). Moreover, p38 MAPK is required in TGF-β1-induced EMT via fibronectin upregulation and collagen expression (7). The published literature therefore strongly supports our hypothesis regarding the requirement of p130cas for TGF-β1-mediated EMT in NSCLC, potentially due to the activation of p38 MAPK.

In addition to p38 activation, TGF-β1 treatment has been shown to upregulate the expression of p-smad2 (29), which is traditionally believed to be an inducer of apoptosis and a tumor suppressor (10,11,30). The present study showed that the levels of p-smad2 peaked following 1 h of treatment with TGF-β1, and then decreased to almost the same level as the controls after 12 h, concordant with the results of other published studies (29–33). The present study indicated that p130cas-knockdown had no marked impact on the activation of Smad2 with or without TGF-β1 treatment. However, in breast cancer cells, p130cas overexpression has been shown to decrease the ability of TGF-β1 to activate Smad2/3, while depleting p130cas led to the increased activation of Smad2/3 (5). We postulate that the mutants of Smad2 in lung cancer A549 cells (34) led to the loss of cross-talk between p130cas and Smad2-activation.

In the present study, p130cas-knockdown had no impact on the expression of cleaved Caspase-3, which is a marker of apoptosis. Our previous study also indicated that the rate of apoptosis following either transient (39.1 vs. 42.1%) or stable transfection (0.33 vs. 0.4%) in A549 cells was not increased by p130cas-knockdown, as observed by FACScan flow cytometry assays (4). All the aforementioned results have indicated that p130cas has no evident association with the apoptosis of A549 cells.

Notably, the p38 MAPK pathway can also induce apoptosis, and inhibitors of p38 MAPK induce anoikis resistance in human breast cancer cells and murine mammary epithelial cells (35,36). The p38 MAPK pathway induces apoptosis and anoikis most likely due to the activation of mitochondrial Bax and subsequent cytochrome *c* release (37). Determining the mechanism by which p130cas blocks p38-rendered apoptosis will be the aim of our future study.

In conclusion, the overexpression of p130cas has significant associations with p-p38 activation *in vivo* and *in vitro*. The present study showed that p130cas had a critical role in TGF-β1-induced EMT in the A549 cells, but no impact on TGF-β1-induced apoptosis. p130cas is a novel molecular ‘rheostat’ that alters the function of the TGF-β1 signaling pathway from tumor suppression to tumor promotion in lung cancer cells.
