# P130cas is required for TGF-β1-mediated epithelial-mesenchymal transition in lung cancer

**DOI:** 10.3892/ol.2020.11416

**Published:** 2020-02-20

**Authors:** Bo Deng, Qun-You Tan, Ru-Wen Wang, Yao-Guang Jiang, Jing-Hai Zhou, Wei Huang

Oncol Lett 8: 454-460, 2014; DOI: 10.3892/ol.2014.2123

Following the publication of this article, the authors have realized that, owing to inadvertent errors during the preparation and revision of the manuscript, an image was misplaced in Fig. 1A. Specifically, the western blotting data shown for the “P38 at 0 h” experiment was selected incorrectly. Additionally, the data showing the ”p-p38 at 1 h” experiment were presented in a distorted and deformed manner in the Figure due to the normalization process of the black background. The corrected version of Fig. 1, featuring all the corrected data for Fig. 1A, is shown opposite.

Note that these changes do not affect the results or the conclusions reported in this paper, and all the authors agree to this Corrigendum (with the exception of Yao-Guang Jiang, who is deceased since the publication of this paper). The authors regret that these errors were allowed to enter into the published version of this article, and apologize to the readership for the inconvenience caused.

## Figures and Tables

**Figure 1.**
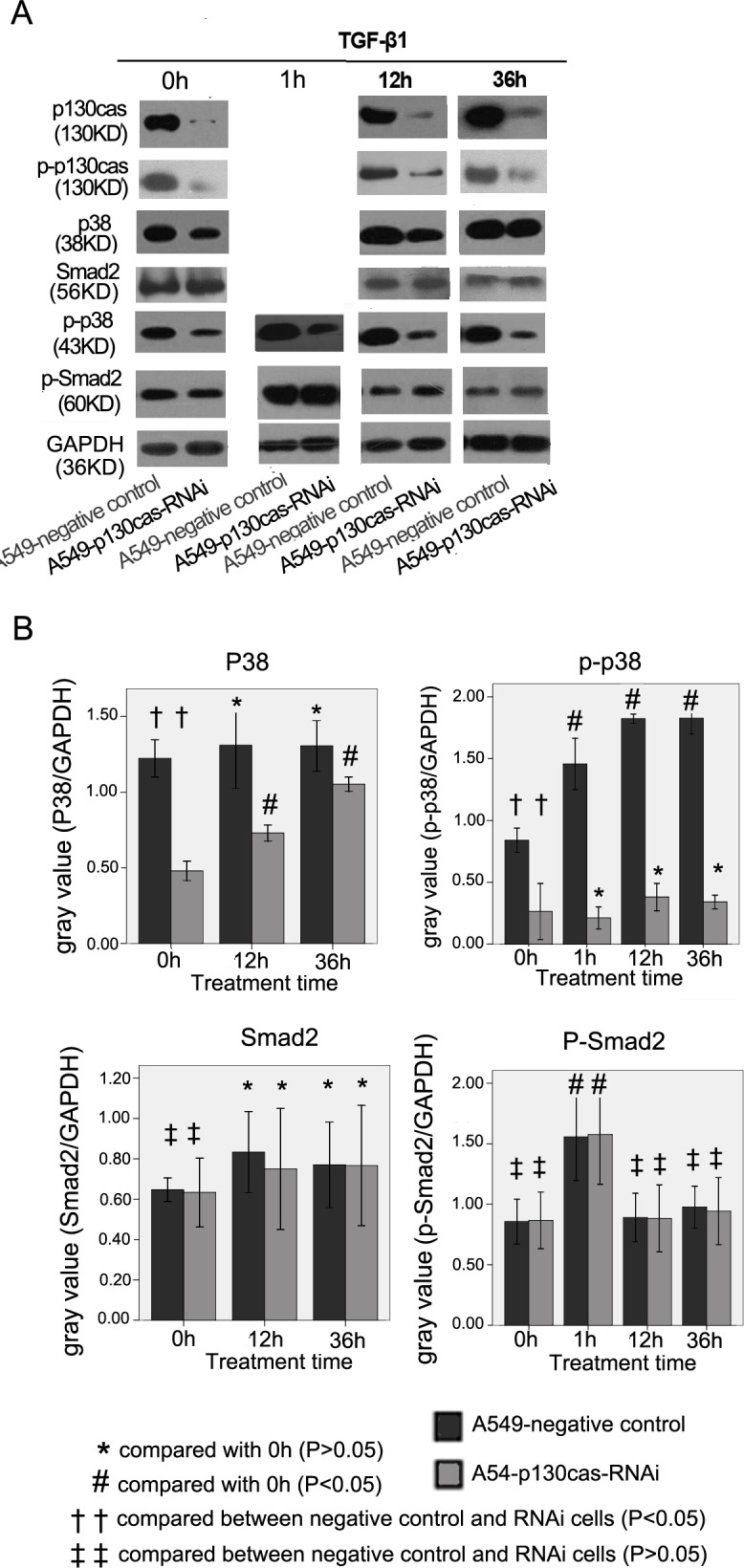
(A) Immunoblotting was conducted to detect the expression levels of p130cas, p-p130cas, p38, p-p38, Smad2 and p-Smad2. The expression levels of p130cas-protein and p-p130cas were reduced markedly in the A549-p130cas-RNAi cells compared with the negative control cells. TGF-β1 treatment (7.5 ng/ml) for 12 h or 36 h markedly increased the p130cas expression levels in the A549 cells. The expression levels of total-p38 and p-p38 were reduced significantly in the A549-p130cas-RNAi cells compared with the A549-negative control cells. The total-p38 expression level was significantly upregulated in the A549-p130cas-RNAi cells following TGF-β1 treatment; however, the p-p38 expression level in the A549-p130cas-RNAi cells was consistently significantly lower than in the A549-negative control cells prior to or following TGF-β1 treatment. TGF-β1 remarkably increased the expression levels of p-Smad2 in the A549 cells following 1 h of treatment. However, p130cas-knockdown had no impact on the expression of total-Smad2 or p-Smad2 compared with the control cells. (B) Quantification of immunoblotting was conducted with Quantity One software. The analyses of the bands of the different proteins were reference against GAPDH. Mean ± SEM values were calculated in order to establish any statistically significant differences. TGF-β1, transforming growth factor-β1; RNAi, RNA interference; cas, Crk-associated substrate.

